# Real‐World Outcomes of Venetoclax and Azacitidine in Hospitalized Patients With Acute Myeloid Leukemia: A Retrospective Study at a Tertiary Care Center

**DOI:** 10.1155/ah/5707397

**Published:** 2026-10-04

**Authors:** Mallory Pane, Durga Bhavani Bandi, Andrew Whiteley, Anushah Mirza, Zachary Gopin, Andrew Irion, Sharon Park

**Affiliations:** ^1^ Department of Internal Medicine Baylor University Medical Center Dallas Texas USA baylor.edu; ^2^ Department of Hematology and Oncology Texas Oncology–Baylor Charles A. Sammons Cancer Center Dallas Texas USA; ^3^ Texas A&M School of Medicine Dallas Texas USA

## Abstract

Venetoclax plus azacitidine improves survival in older or unfit patients with newly diagnosed acute myeloid leukemia (AML), as demonstrated in the VIALE‐A trial. ^2^ However, real‐world data in hospitalized patients—particularly those with secondary AML or adverse‐risk mutations—remain limited. We conducted a retrospective study of exclusively hospitalized patients with treatment‐naïve AML who completed at least one full 28‐day cycle of azacitidine and venetoclax at Baylor University Medical Center between October 2019 and October 2024, with the aim of evaluating outcomes at a clinically relevant treatment inflection point. Risk stratification was performed using ELN 2022 criteria, and safety was assessed using CTCAE v5.0. Fifty‐six patients met inclusion criteria (median age 71 years); 51.8% had secondary AML, and 53.7% had adverse‐risk disease. Among 43 evaluable patients, the composite complete remission (CRc) rate after one cycle was 55.8%. Median overall survival (OS) was 17.6 months. Patients with TP53 mutations had significantly shorter median OS (3.8 months) compared with those without TP53 mutations (20.2 months). Common grade ≥ 3 toxicities included leukopenia (67.8%) and febrile neutropenia (57.6%). By focusing on patients who completed an initial treatment cycle, this study provides conditional survival estimates that may inform decisions regarding continuation of therapy. Despite the predominance of secondary and adverse‐risk AML in this cohort, OS was comparable to that observed in VIALE‐A, supporting the effectiveness of azacitidine plus venetoclax in hospitalized AML patients ineligible for intensive chemotherapy.

## 1. Background

Acute myeloid leukemia (AML) carries a poor prognosis, particularly in older patients and those with significant comorbidities who are ineligible for curative‐intent intensive chemotherapy [1].

The combination of azacitidine and venetoclax has been shown to improve outcomes compared with single‐agent azacitidine in treatment‐naïve AML patients who are not candidates for intensive therapy [2]. However, some single‐institution studies have suggested that real‐world outcomes may be inferior to those observed in controlled clinical trials [3]. This study was designed to evaluate the real‐world safety and efficacy of azacitidine plus venetoclax in exclusively hospitalized AML patients treated in a large tertiary community hospital.

## 2. Methods

### 2.1. Study Design and Population

We conducted a retrospective chart review at Baylor University Medical Center to evaluate real‐world outcomes of azacitidine plus venetoclax as first‐line therapy in hospitalized AML patients deemed poor candidates for intensive chemotherapy. This analysis was specifically designed to assess outcomes among patients who were able to tolerate and complete a full first 28‐day cycle of azacitidine and venetoclax, thereby evaluating survival and response in a selected cohort distinct from prior real‐world studies that include patients unable to complete initial therapy.

Eligible patients had a confirmed diagnosis of AML, were hospitalized between October 30, 2019, and May 31, 2024, and completed at least one full 28‐day cycle of azacitidine and venetoclax by. All patients received venetoclax for 28 days and azacitidine for 7 days per cycle. Patients were excluded if they did not complete one full cycle or had received prior outpatient venetoclax. A total of 56 hospitalized patients met inclusion criteria.

Posttreatment bone marrow biopsies at approximately day 30 were available for 43 patients, who were evaluable for response assessment. Thirteen patients did not undergo posttreatment biopsy, most commonly due to clinical instability or transition to comfort‐focused care; these patients were included in survival and safety analyses but not response evaluation.

Somatic mutations (IDH1, IDH2, FLT3‐ITD, FLT3‐TKD, ASXL1, NPM1, and TP53) were recorded when testing was available. ELN 2022 risk stratification was applied. Two patients lacked complete diagnostic marrow specimens and were excluded from ELN 2022 molecular risk categorization and subgroup analyses. Adverse events (AEs) were graded using CTCAE v5.0.

### 2.2. Definitions and Statistical Analysis

Response was assessed after one cycle using bone marrow biopsy. Complete remission (CR) was defined as < 5% blasts with ANC ≥ 1000/μL and platelets ≥ 100,000/μL. CR with incomplete count recovery (CRi) was defined as < 5% blasts without full count recovery. Composite complete remission (CRc) was defined as CR + CRi. Overall survival (OS) was estimated using Kaplan–Meier methods, including subgroup OS analyses for patients with secondary AML and those with a TP53‐mutation.

### 2.3. Ethics Statement

This retrospective study, “Retrospective Study to Evaluate the Real‐world Outcomes of Frontline Venetoclax with Azacitidine in Hospitalized AML Patients at a Major Tertiary Center” (Reference Number: 408872), was reviewed and approved by the Baylor Scott and White Research IRB Red (Protocol #024‐566) on January 20, 2025. The approval period extends through January 19, 2026, with the next continuing review scheduled for December 19, 2025. The study was approved to be conducted at Other Baylor Facility. Key study personnel included Mallory Pane, MD, and Andrew Whiteley, MD. The IRB granted a waiver of informed consent and authorization in accordance with 45 CFR 46.116(f) and 45 CFR 164.512 (i) (2) (ii), determining that the research involved no more than minimal risk, could not practicably be conducted without the use of existing protected health information, and that only deidentified data are reported.

All authors have read and approved the final version of the manuscript. Mallory Pane, MD, had full access to all of the data in this study and takes complete responsibility for the integrity of the data and the accuracy of the data analysis.

## 3. Results

### 3.1. Patient Characteristics

Between October 2019 and May 2024, 416 AML patients were admitted to Baylor University Medical Center; 107 received azacitidine plus venetoclax, and 56 patients met inclusion criteria.

Median age was 71 years (range 25–83). Twenty‐nine patients (51.8%) had secondary AML, including 21 with prior hematologic disorders, 3 with AML with myelodysplasia‐related changes, and 5 with therapy‐related AML. Among patients with secondary AML arising from antecedent MDS, CMML, or MDS/MPN, none had received prior hypomethylating agent therapy before the initiation of azacitidine and venetoclax for AML. Twenty‐nine patients (53.7%) were classified as adverse‐risk by ELN 2022 criteria.

Baseline bone marrow blasts were < 30% in 19 patients (33.9%), 30%–49% in 15 patients (26.8%), and ≥ 50% in 20 patients (35.7%); blast percentage was unavailable in two patients (3.6%).

Somatic mutations included TP53 in 15 patients, IDH1/IDH2 in 12, ASXL1 in 10, and FLT3 in 10 (8 FLT3‐ITD and 2 FLT3‐TKD). Several patients harbored multiple mutations. Baseline characteristics are summarized in Table 1.

**TABLE 1:** Baseline characteristics.

Characteristic	Value
Median age, years (range)	71
Sex, *n*	
Male	33
Female	23
AML Type, *n*	
De novo AML	27
Secondary AML	29
AML with MDS‐related changes	3
Prior MDS, CMML, or MDS‐MPN	21
Therapy‐related AML	5
Bone marrow blast count, *n*	
< 30%	19
30%–49%	15
≥ 50%	20
Unavailable	2
ELN risk category, *n* = 54	
Favorable	7
Intermediate	18
Adverse	29
Somatic mutations, *n*	
TP53	15
IDH1/IDH2	12
ASXL1	10
FLT3 (any)	10
FLT3‐ITD	8
FLT3‐TKD	2

### 3.2. Efficacy

Among 43 evaluable patients, CR was achieved in 8 (18.6%), CRi in 16 (37.2%), yielding a CRc rate of 55.8%. Nineteen patients (44.2%) were nonresponders after one cycle (Table 2). All patients who achieved CR or CRi were evaluated for allogeneic hematopoietic stem cell transplantation (HSCT). Five patients proceeded to allogeneic HSCT following treatment with azacitidine and venetoclax, representing 11.6% of the patients achieving CR/CRi. Among responding patients who did not proceed to transplant, HSCT was not pursued because patients were either deemed transplant‐ineligible by the treating/transplant team based on clinical factors or declined HSCT after discussion with their treating physicians. Relapse occurred in 4 patients overall, including 1 patient with de novo AML and 3 patients with secondary AML arising from antecedent MDS, CMML, or MDS/MPN. Among patients who relapsed, the duration of response was 288 days for the patient with de novo AML and 35, 82, and 94 days for the patients with secondary AML, respectively.

**TABLE 2:** Response to azacitidine plus venetoclax therapy (*n* = 43 evaluable patients).

Response	*n* (%)
Complete remission (CR)	8 (18.6)
CR with incomplete recovery (CRi)	16 (37.2)
Composite CR (CR + CRi)	24 (55.8)
Non‐response after one cycle	19 (44.2)

Median OS for the overall cohort was 17.6 months (Figure 1). Median OS was 17.6 months for secondary AML versus 13.7 months for de novo AML (Figure 2). TP53‐mutated AML was associated with markedly inferior OS (3.8 months vs. 20.2 months; Figure 3).

**FIGURE 1:**
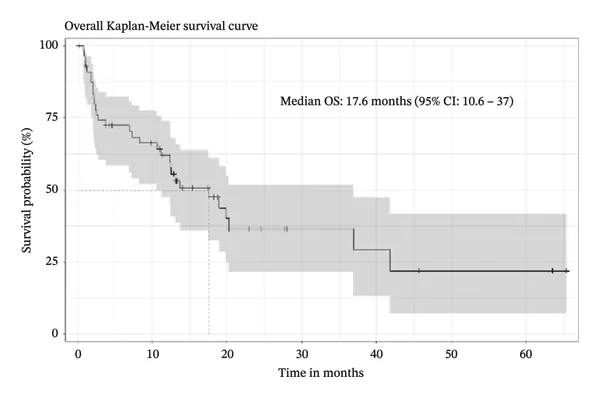
Overall Kaplan–Meier survival curve.

**FIGURE 2:**
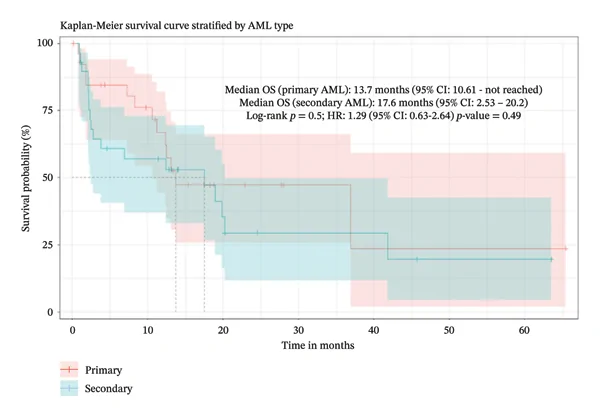
Kaplan–Meier survival curve stratified by AML type.

**FIGURE 3:**
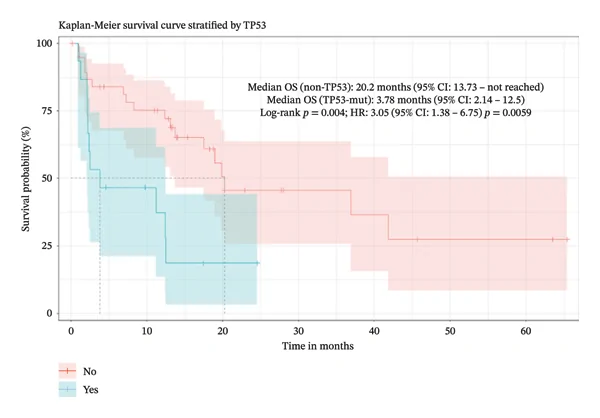
Kaplan–Meier survival curve stratified by TP53.

### 3.3. Safety

AEs were common, with 54 patients (96.4%) experiencing at least one AE and 47 patients (83.9%) experiencing grade ≥ 3 toxicity. The most frequent grade ≥ 3 hematologic toxicities were leukopenia (71.4%), thrombocytopenia (64.3%), and febrile neutropenia (60.7%).

The most common nonhematologic AEs were infection without sepsis (35.7%), pneumonia (26.8%), and acute kidney injury (26.8%). Detailed AEs are summarized in Table 3. Early mortality (within 60 days) occurred in 7 patients (12.5%). Causes of death are listed in Table 4.

**TABLE 3:** Adverse events during azacitidine and venetoclax treatment (*n* = 56).

Adverse event	All grades, *n* (%)	Grade ≥ 3, *n* (%)
Any adverse event	54 (96.4)	47 (83.9)
Leukopenia	48 (85.7)	40 (71.4)
Neutropenia	46 (82.1)	22 (39.3)
Thrombocytopenia	43 (76.8)	36 (64.3)
Anemia	40 (71.4)	21 (37.5)
Febrile neutropenia	34 (60.7)	34 (60.7)
Infection without sepsis	20 (35.7)	7 (12.5)
Pneumonia	15 (26.8)	7 (12.5)
Acute kidney injury	15 (26.8)	6 (10.7)
Sepsis	8 (14.3)	4 (7.1)
Other infections	5 (8.9)	0 (0.0)

**TABLE 4:** 60‐day mortality and cause of death.

Patient	Cause of death
Patient 6	Refractory AML
Patient 19	Refractory AML
Patient 31	Cardiac arrest
Patient 35	Infectious cause
Patient 40	Respiratory failure
Patient 50	Respiratory failure
Patient 53	Refractory AML

## 4. Discussion

In this retrospective, real‐world analysis of 56 hospitalized AML patients treated with azacitidine and venetoclax, more than half had adverse‐risk disease by ELN criteria, most had secondary AML, and approximately one in four had a TP53 mutation. Despite these high‐risk features, the CRc rate after one cycle was 55.8%, exceeding the 43.4% reported in VIALE‐A [2]. The median OS of 17.6 months was comparable to the 14.7 months observed in VIALE‐A, supporting the effectiveness of this regimen in high‐risk hospitalized patients. Consistent with prior studies, patients with TP53‐mutated AML had a markedly poor prognosis, with a median OS of only 3.8 months, highlighting the continued unmet therapeutic need in this subset of patients [4, 5].

Prior real‐world studies evaluating azacitidine and venetoclax in AML have largely included unselected patient populations, encompassing individuals who experience early clinical deterioration or are unable to complete an initial treatment cycle. While such analyses provide important insights into treatment tolerability and early mortality, they may obscure prognostic information relevant to patients who successfully complete therapy. Our study was intentionally designed to focus on a selected cohort of patients who were fit enough to complete a full first cycle of azacitidine and venetoclax, allowing for a more refined assessment of outcomes with reduced confounding from baseline factors that may limit tolerance of therapy in general.

By restricting the analysis to patients who completed one full cycle of azacitidine and venetoclax, our study provides conditional, or “actuarial,” OS estimates for a clinically relevant subgroup—patients who demonstrate hematologic response yet fail to achieve meaningful improvement in functional status. This addresses an important unmet need in the community by offering clinicians prognostic information to guide decision‐making in patients who exhibit discordance between disease response and clinical recovery after cycle one. Completion of the initial treatment cycle thus represents a meaningful inflection point in AML management that can inform expectations and subsequent therapeutic decisions.

In this context, our findings reinforce that treatment decisions following completion of cycle 1 of azacitidine and venetoclax should integrate underlying disease biology and functional status, rather than short‐term treatment tolerance alone. For example, in patients with adverse‐risk disease, particularly those harboring TP53 mutations, OS remains poor despite successful completion of an initial treatment cycle without major complications. Among these patients, persistent poor performance status after cycle 1—especially when prolonged hospitalization precludes return to baseline functional independence—suggests a limited likelihood of meaningful survival benefit from additional therapy, and continuation of treatment may not be warranted. In contrast, in patients with relatively favorable‐ or intermediate‐risk AML who continue to have impaired performance status after cycle 1, continuation of therapy—including prolonged inpatient management for subsequent cycles—may be justified given the potential for durable clinical benefit seen in this study. Overall, by focusing on patients who complete cycle 1 of azacitidine and venetoclax, our study provides conditional survival estimates that clarify how disease biology and functional recovery—rather than short‐term treatment tolerance alone—should guide decisions about continuing therapy.

Another notable finding in our study was that secondary AML patients constituted over half of our cohort—more than double the proportion in VIALE‐A—yet experienced OS comparable to de novo AML patients. The reason for this preponderance is not evident, but our secondary AML patients experienced a similar OS to the de novo AML patients, consistent with findings from VIALE‐A [2]. Notably, no patients with secondary AML arising from antecedent myeloid neoplasms had received prior hypomethylating agent therapy, aligning this subgroup with VIALE‐A eligibility criteria and strengthening the relevance of comparisons between our secondary AML cohort and the VIALE‐A secondary AML population. However, relapse was numerically more frequent among patients with secondary AML, and this finding should be interpreted cautiously given the small number of relapse events, limited subgroup size, and selected nature of this cohort, which included only patients who completed at least one full cycle of therapy. Together, these findings providereal‐world support for the effectiveness of azacitidine and venetoclax in secondary AML patients who are unfit for intensive chemotherapy.

Historically, outcomes with intensive chemotherapy in secondary AML have been poor. CPX‐351 demonstrated improved OS compared with 7 + 3, but survival remained limited [6]. Moreover, prior data suggest that hypomethylating agents alone offer OS comparable to intensive chemotherapy in older or secondary AML populations, respectively [7, 8]. Given that azacitidine plus venetoclax improves both response rates and OS compared with azacitidine alone [2], our findings support the hypothesis that secondary AML patients may derive greater benefit from this combination than from intensive chemotherapy.

Although azacitidine and venetoclax are often selected for older or comorbid patients who are ineligible for intensive chemotherapy, our findings also highlight its potential role as a bridge to curative‐intent therapy in select patients. In our cohort, five of the 43 response‐evaluable patients (11.6%) proceeded to allogeneic HSCT after treatment with azacitidine and venetoclax, and four patients remain long‐term survivors following transplant. Importantly, all patients who achieved CR or CRi were evaluated for allogeneic HSCT, and HSCT was not pursued in the remaining responders due to transplant ineligibility as determined by the transplant team or declined HSCT after discussion with their treating physicians. Most transplanted patients were in CR at the time of HSCT, apart from one patient who nonetheless achieved long‐term survival of 5 years after transplant. These observations suggest that, in appropriately selected patients who achieve disease control and maintain adequate functional status, azacitidine and venetoclax may provide a pathway to definitive consolidation with HSCT rather than serving solely as palliative therapy.

## 5. Limitations

Limitations include the retrospective design, single‐center setting, modest sample size, and limited follow‐up. Because the cohort consisted exclusively of hospitalized patients, generalizability to patients treated primarily in the outpatient setting may be limited. Molecular testing was incomplete in a small number of patients; two individuals lacked complete diagnostic marrow specimens and were excluded from ELN 2022 molecular risk categorization and molecular subgroup analyses, which may affect the robustness of ELN‐based risk stratification and subgroup outcome interpretation. Additionally, response assessment after one cycle likely underestimates best response, as some patients achieve remission after subsequent cycles. Despite these limitations, a key strength of this study is its focus on a clinically selected cohort of hospitalized AML patients, including a substantial proportion with secondary and adverse‐risk disease, who completed an initial treatment cycle, offering decision‐relevant survival estimates that complement prior real‐world analyses.

## 6. Conclusion

In this real‐world analysis of hospitalized patients with AML treated with azacitidine and venetoclax, meaningful clinical activity was observed despite a high prevalence of adverse‐risk disease, secondary AML, and TP53 mutations. OS was comparable to that reported in VIALE‐A, and secondary AML patients experienced outcomes similar to those with de novo disease, supporting the effectiveness of this regimen in patients ineligible for intensive chemotherapy.

Importantly, by focusing on patients who completed a full first cycle of therapy, this study provides conditional survival estimates that inform treatment decisions at a clinically relevant inflection point. These findings highlight that disease biology and functional recovery following cycle 1—rather than short‐term treatment tolerance alone—should guide decisions regarding continuation of therapy. In select patients, azacitidine and venetoclax may also serve as a bridge to curative‐intent transplantation, underscoring its potential role beyond a purely palliative strategy.

## Funding

No funding was received for this manuscript.

## Conflicts of Interest

The authors declare no conflicts of interest.

## Data Availability

The data that support the findings of this study are available from the corresponding author upon reasonable request.
